# Medical and Philosophical Intelligence

**Published:** 1812-07

**Authors:** 


### MEDICAL AND PHILOSOPHICAL INTELLIGENCE.

*GEOLOGICAL SOCIETY.—April* 3.—A notice relative to the geology of the coast of Labrador, by the Rev. Mr. Steinhauer, was read.

The only accounts that have been hitherto published concerning this part of the British dominions are the Memoir of Mr. (afterwards Sir Roger) Curtis, inserted in the Philosophical Transactions, and Mr. Cartwright's Journal.

The Moravian Missionaries, in 1772, established in this country their first settlement, called Nain, in lat. 56° 38′; and subsequently Okkak, in lat. 58° 43′; and Hopedale, in lat. 55° 36′. In the course of the last year they doubled Cape Chudleigh, in lat. 60° 20′, and descended on the western side of the same promontory as far as lat. 58° 36′.

The leisure of the Missionaries, when opportunities occur, is employed in collecting materials for a natural history of the country; they have kept tables of the thermometrical and barometrical variations, have procured specimens of most of the native vegetable productions, and have from time to time sent over specimens of such minerals as attracted their notice.

The general aspect of this dreary region as that of bare and barren rock towering in craggy eminences, and of sandy marshes, on which are found a few pines and brushwood and aquatic mosses. In several parts of the country the rocks are intersected by chasms, running generally in a right line to a considerable distance, which when covered with snow form dangerous pitfalls. The highest mountains extend along the eastern coast: the elevation of one of them, called Mount Thoresby, has been ascertained by actual measurement to equal 2733 feet, and a few others probably attain the height of 3000 feet.

From the islands near Cape Chudleigh the Missionaries have sent specimens of large-grained pale granite with garnets. The island of Ammitok, in lat. 59° 20′, consists entirely of a crumbling garnet rock, in which hornblende sometimes occurs. The mountains about Nachwak Bay furnish lapis ollaris.

On the south of the high land of Kiglapyed, in lat. 57°, the district commences where the Labrador felspar is found: this mineral occurs not only in rolled stones on the shore, but in spots on the rocks, rocks in the neighbourhood of Nain, and particularly in the rocks bordering a lagoon about 60 miles inland, in which Nain North river terminates. The same district also produces the hyperstene or Labrador hornblende.

At Hopedale a limestone occurs from which have been procured specimens of reddish limestone, of calcareous spar, and of a variety of schiefer spar.

The country to the west of Cape Chudleigh, as far as it has been explored, is called the Ungava; and abounds with red jasper, with hæmatites, and with iron pyrites.

*April* 17.—An account of the brine springs at Droitwich, by Leonard Horner, Esq. Sec. Geol. Soc. was read.

The town of Droitwich has been noted for the manufacture of salt during at least a thousand years; but no detailed account has hitherto been published of the natural and chemical history of the brine springs from which it is procured. The brine springs are in the centre of the town, being situated in a narrow valley through which the small river Salwarp flows. The prevailing rock about Droitwich is a fine-grained calcareo argillaceous sandstone of a brownish red color, with occasional spots and patches of a greenish blue. At Doder-hill, in the immediate vicinity of the salt pits, the rock appears to be a stratified sandstone of a greenish grey color, and more indurated than the red rock. It also differs from this last in containing slender veins of gypsum.

No new brine pits have been sunk for the last thirty years: the only particulars therefore concerning the strata covering the salt, which Mr. Horner has been able to obtain, are derived from Dr. Nash's History of Worcestershire, and from an inhabitant of Droitwich who was on the spot when the last pit was sunk. From these authorities it appears, that the depth of from 35 to 45 feet below the surface is occupied by beds of gravel, of red marly clay, and of blue and white stone. To these succeeds a bed of gypsum about 105 feet in thickness, immediately below which is what is called the *River of Salt;* which is a stratum of nearly saturated brine of 22 inches in depth, lying on a bed of rock salt, the thickness of which is unknown, no borings have been sunk in it to a greater depth than five or six feet. In constructing the pits, the method is to sink a shaft about eight feet square into the gypsum, and then to pierce this bed by a borer four inches in diameter: the borer is known to have passed through the gypsum by its suddenly dropping 22 inches, the depth of the River of Salt. As soon as the borer is withdrawn, the brine suddenly rushes up and overflows at the mouth of the pit.

There are only four pits at present in use, and the annual quantity of salt which they afford is about 16,000 tons.

The brine from all the pits is perfectly limpid, and when in a large body has a pale greenish hue similar to that of sea-water. To the taste it is intensely saline, but without any degree of bitterness. The specific gravity differs in the different pits, probably on account of the greater or less accuracy with which the land springs are stopped out: that of perfectly saturated brine is equal to 1210·39

water

(water being 1000); that of the five pits examined by Mr. Horner was found to vary from 1206·11 to 1174·71; and an evaporation afforded from 2289·75 grs. to 1922·97 grs. of entire salt, dried at 180° Fahr., in a pint.

This salt, from a careful analysis, appears to be composed of

| | |
|---|---|
| 96·48 | muriate of soda. |
| 1·63 | sulphate of lime. |
| 1·82 | sulphate of soda. |
| 0·07 | muriate of magnesia. |
| 100·00 | |

On comparing the brine of Droitwich with that of Cheshire, as described by Mr. Holland in his Agricultural Survey of that county, and by Dr. Henry, in his paper on the subject in the Philosophical Transactions, it appears that the strength of the different brines is nearly the same; that the Cheshire brine contains rather a larger proportion of muriate of soda; that the Droitwich brine is free from carbonate of lime, oxide of iron, and muriate of lime, all of which are found in the Cheshire brine; and finally, that the latter is free from the sulphate of soda which is contained in the former.

---

*Wernerian Natural History Society.*—At the meeting of this Society on the 7th of March, the Secretary read an "Essay on Sponges, with descriptions of all the species that have been discovered on the coast of Great Britain," by George Montagu, esq. of Devonshire. From Mr. Montagu's researches, as to the constitution of sponges, it appears that no polypi or vermes of any kind are to be discerned in their cells or pores: they are, however, decidedly of an animal nature; but they possess vitality without perceptible action or motion. Mr. Montagu has divided the genus Spongia into five families, viz. branched, digitated, tubular, compact, and orbicular. Only fourteen species were previously known to be British. Mr. Montagu in this communication described no fewer than thirty-nine. A considerable number of the species are quite new, or have now for the first time been distinguished and formed by that indefatigable naturalist.

At the same meeting Dr. Yule read a memoir on the natural method in botany, in which he defended the existence of the series of natural affinity in plants against objections of Professor Willdenow and Dr. Smith, founded on the want of regularity in the series, &c. He contended, that the illustrious author of the artificial system never intended that it should supersede, but, on the contrary, that it should lay the foundation of, the natural classes, "quas plana genera nondum detecta revelabunt:" and that with this view he uniformly inculcated the study of natural genera, in conformity with his great maxim, "Omne genus naturale."

---

*Russell Institution.*—In Mr. Bakewell's third lecture, he noticed the fractures and fissures in the earth's surface, which are filled with mineral substances differing in quality from the rocks or strata they intersect. In travelling over an extensive range in a mountainous country, he observed that the form and inclination of the hills may present the same general resemblance for a considerable distance; but we may sometimes perceive that in certain situations the same kind of rock is thrown into a different position, and the strata are inclined in an opposite direction. When such a change of position occurs, then we may be almost certain that a rent or fissure of the earth's surface has taken place, and that the continuity of the strata has been broken by some cause which has elevated one side and depressed the other. These rents are called by miners dykes or faults. In the northern part of our island, they are frequently filled with basalt or whinstone. In the coal districts of Yorkshire, and in the midland counties, they are more frequently filled with clay. In some situations, where these dykes occur in contact with coal, the coal appears charred on each side. He stated instances of this in the county of Durham, and in Shropshire. Such phenomena appear to indicate that the basalt had been ejected into the fissure in a state of igneous fusion.

The length to which dykes extend has in a few instances been traced, owing to the upper part being hid by a covering of loose earth or soil. They vary in width, from 6 inches to 70 feet or more. Their depth remains unascertained. Veins of granitic and other rocks occur also amongst primary mountains.

Metallic veins resemble dykes in their structure, but not in their contents, being filled with different metallic substances in a state of ore, and intermixed with various crystals and earths. Mr. Bakewell described the contents of many remarkable veins in England and other parts of the world, and the extent and depth to which they have been traced. He explained their inclination, position, and structure, by various drawings and specimens. It appeared from his description, that the Eton copper-mine has been worked to a greater depth than any other mine in England, ore having been lately got at the depth of 236 fathoms, or 472 yards. Mr. Bakewell described the various states in which the same metal is sometimes found in one mine, as native metal, or as alloyed with other metals, or mineralized by oxygen, sulphur, or acids.

The lecturer proceeded to observe, that the question "In what manner have metallic veins been filled with their contents?" has greatly divided the opinions of geologists. Dr. Hutton supposed that mineral and metallic veins have been formed by the breaking of the solid rocks from the expansive force of subterranean fires, and their contents ejected into them in a state of fusion from below. Mr. Werner supposes they are fissures produced by the shrinking or drying of the materials of which rocks are composed, and afterwards filled from above by the metallic matter in a state of solution. Both these theories, Mr. Bakewell observed, were at variance with the appearances which mineral veins present.

The favorable reception which Mr. Werner's system of the formation of metallic veins has met with, was, he said, a striking instance of the extent to which an attachment to theory can be carried, and close the eyes of its votaries to the plainest and most obvious obvious facts. Had metallic veins been filled from above, by the metallic ores infused in a state of solution, this solution must at one time have covered the whole earth, and the ores deposited by it would have filled all the valleys and cavities on the earth's surface, and would not have been confined to the narrow fissures or veins in which they are found. There are, however, he observed, existing facts equally decisive against the theories of Dr. Hutton and Mr. Werner respecting the formation of veins. It is almost universally found, that, where a metallic vein passes through a mountain composed of beds or strata of different kinds of rock, the nature or quality of the ore varies in each kind of rock. This is the case in Cornwall, where the same vein traverses grau-wacke or killas and granite, it is found to be more rich in the latter rock. In Derbyshire, the veins of galena which pass through the limestone rocks disappear in the basaltic amygdaloid by which the lime rocks are separated; or, if the vein passes through the amygdaloid, it very rarely contains any quantity of metallic ore. It is also found that different strata or layers in the same bed of lime rock are more productive in ore than others. If metallic veins were filled by infusion from above, or ejected from below, it is impossible that the nature of the rocks they traverse could alter the quality or quantity of their products. This fact, though undeniable, has never, Mr. B. observed, been properly attended to, and is alone decisive against the received opinions respecting the formation of metallic veins, and seems to prove that the rock itself had been in some manner operative in the production of their contents.

Mr. B. observed, that the present state of chemical science did not admit us to form any decisive or satisfactory opinion on this subject, and he considered it much wiser to acknowledge our ignorance, than frame theories which only serve to perpetuate error. It is not at present fully ascertained, whether metals be simple substances or compounds: but this discovery, he said, must precede the formation of such a theory as will explain in a satisfactory manner many of the mysteries of the mineral kingdom.

Some experiments which he showed, offered, he said, hints to future discoveries on this subject. All the metals are capable of existing in peculiar solutions, and some of them (perhaps all) are also capable of becoming elastic invisible gases when united with hydrogen, and can be made to deposit the metallic contents in a solid form. In this manner they may have entered veins, and may have been separated from the substance of rocks, and decomposed by a process analagous to that of the Voltaic electricity, the different sides of the vein acting like the opposite ends of the pile.

Mr. Bakewell observed, that much yet remained to be done to improve the processes of metallurgy, and, what was of more importance, to provide for the health and safety of the persons who labour in mines and the reduction of ores. The immense annual sacrifice of lives in the mines of Peru is a melancholy instance of the baneful effects resulting from ignorance, tyranny, and avarice. In Mexico, according to late observation, the condition of the miners is much improved. Science, Mr. B. observed, had ever been found the friend of man; and when she shall dawn on those regions, her beneficent effects will soon be felt: she will teach him to supply subterranean works with salutary currents of air, and extend his researches with safety to far greater depths than have yet been explored. Scientific pursuits are not, as some may imagine, unprofitable speculations: they have always a tendency to enlarge the sphere of human power, to add to our comforts, and lessen the evils of suffering humanity.

---

We have been favored by Jesse Foot, esq. with an extract of a letter from his nephew, Mr. J. Foot, dated Clarendon, Jamaica, March 13, 1812, stating his success with the cow-pox, in upwards of two hundred patients, whom he inoculated with it in the preceding March and April; and whom also he inoculated with small-pox in December last. He began by infecting two children of color, from a subject that had the disease in high perfection, from matter sent out by the Vaccine Society in London, to a medical friend in the same district; they both took, and fevered at the usual period, and from them fourteen negro children were inoculated. These also fevered at the usual time, and thus Mr. F. continued through his practice, taking one estate at a time, by which means he was enabled to keep up a source for fresh matter, and to pay more particular attention to the progress of the disease than he would otherwise have been able to have done. He had great trouble to prevent their rubbing off the pustule, which sometimes hindered the disease from shewing its genuine appearance.

The small-pox was brought to the parish in December, and of course afforded an opportunity of confirming Mr. Foot's exertions with the test, which he did by inoculating all the patients whom he had previously vaccinated, from a small-pox subject. The result was, that only two, out of two hundred and forty, took the small-pox.

"Several children that were born from April to December, were also inoculated with the small-pox; at the same time they all had eruptions, and were allowed to have free intercourse with the others, so that the former resisted the variolous disease, not only from inoculation, but by effluvia also. In addition to the above, on one estate, where upwards of twenty children had been vaccinated, an adult broke out with the confluent, natural small-pox, and died."

The gentleman from whom Mr. F. obtained his vaccine matter was equally successful as himself. This information is the more gratifying, because we have understood from some intelligent planters, that there has been considerable difficulty in inducing the medical practitioners, on most of the estates, to inoculate with vaccine instead of variolous matter.

---

Mr. Crowther requests us to announce, that he has drawn up a brief reply "to the illiberal perversions which certain reviewers have thought fit to make of the plain common sense contained in his publication on insanity." The statement may be had, gratis, on applying to Mr. Crowther, or to Mr. Underwood, Medical Bookseller.

*Catalogue*

*Catalogue of the Professors and Students of the Medical Institution at Fairfield, Herkimer County, N. Y. Oct.* 1811.

Josiah Noyes, M.B. Professor of Chemistry, Mineralogy, and Materia Medica.

Lyman Spalding, M.D. C.S.M.S. Lond. Professor of Anatomy and Surgery.

Geo. Cheyne Shattuck, M.D. Professor of the Institutes of Medicine.

Dr. Westel Willoughby, Professor of Obstetrics.

| *Names.* | *Towns.* | *Counties.* |
|---|---|---|
| William H. Averill, | Cooperstown, | Otsego, *N. Y.* |
| Jonathan Bailey, | Weare, | Hillsboro', *N. H.* |
| Thomas Baker, | Mamakating, | Orange, *N. Y.* |
| Stephen C. Barnum, | Southeast, | Dutchess. |
| Elijah Barnum, | Rensselaerville, | Albany. |
| S. D. W. Bloodgood, | Utica, | Oneida. |
| Benjamin Bowen, jun. | Newport, | Herkimer. |
| Caleb Budlong, | Frankfort, | ditto. |
| J. L. C. Cazier, | Lebanon, | Madison. |
| Thomas L. Conkling, | Rensselaerville, | Albany. |
| Samuel H. Davis, | New York, | New York. |
| David Dickerson, | Fairfield, | Herkimer. |
| Ab'm Diefendorf, | Canajohary, | Montgomery. |
| Samuel G. Dorr, | Chatham, | Columbia. |
| Charles E. Ford, | Ogdensburgh, | St. Lawrence. |
| Alden Gage, jun. | Fairfield, | Herkimer. |
| Henry Green, | ditto, | ditto. |
| Alpheus S. Green, | Mewport, | ditto. |
| J. H. Gregory, | Milton, | Saratoga. |
| James Hadley, | Weare, | Hillsboro', *N. H.* |
| William F. Haile, | Fairfield, | Herkimer, *N. Y.* |
| David M. Hale, | Herkimer, | ditto. |
| John M. Henderson, | Champion, | Jefferson. |
| Henry Jeffers, | Fairfield, | Herkimer. |
| Uriel H. Kellogg, | Whitestown, | Oneida. |
| William Lincoln, | Fitzwilliam, | Cheshire, *N. H.* |
| Hiram Mann, | Milton, | Saratoga. *N. Y.* |
| Nathan H. Manter, | Litchfield, | Herkimer. |
| Horace Manley, | Norway, | ditto. |
| James M'Master, jun. | Russia, | ditto. |
| Alonzo H. Maynard, | Herkimer, | ditto. |
| Jared S. Maynard, | ditto, | ditto. |
| Timothy Miller, | Turin, | Lewis. |
| William M. Millard, | Milton, | Saratoga. |
| Adams W. Platt, | Galway, | ditto. |
| Alex. Richards, jun. | Madrid, | St. Lawrence. |
| David N. Schanck, | Middletown, | Monmouth, *N. J.* |
| James Sheldon, | Remsen, | Oneida, *N. Y.* |
| Ira Smith, | Ogdensburgh, | St. Lawrence. |
| Willard Smith, | Lewistown, | Niagara. |
| Nathaniel Strong, | Herkimer, | Herkimer. |
| John M. Watson, | Newport, | ditto. |
| Newell Wright, | Ballston, | Saratoga. |

*An*

*An Account of the Symptoms and successful Treatment of a Person who swallowed a large Quantity of Laudanum.*—A young woman, 20 years of age, on the 15th of July, 1811, took 10 drachms of laudanum about eleven o'clock in the forenoon, which was not discovered till near two in the afternoon of the same day. The whole of the laudanum had been retained on her stomach, notwithstanding she had taken a strong solution of the sulphate of zinc. When I first saw her, about three o'clock, she appeared much convulsed, her hands and jaws were firmly closed, her countenance of a ghastly paleness, and she was totally insensible. As the sulphate of zinc had produced no effect, a solution of the sulphate of copper with some ipecacuanha was given every ten minutes until vomiting was produced. The temples and nostrils were constantly irritated with hartshorn, and after she had taken about three doses of the solution, vomiting came on, which in some degree roused her. By the assistance of two persons she was kept constantly walking or rather dragged about the room, and the operation of the emetic was promoted by draughts of warm water. Not the slightest smell or appearance of laudanum could be perceived in the fluid that was ejected. She complained of insupportable drowsiness, and weakness in her knees, urging her inability to walk, and requesting that she might be allowed to sleep. The emetic was repeated, but no laudanum was rejected. When the action of the emetic had ceased, I caused her to drink a considerable quantity of vinegar and water, still keeping her walking or in a state of agitation. At length she became less drowsy, and towards evening could walk with little assistance, but appeared very much exhausted. A mixture with ammonia was now given, and she was permitted to lie down in bed, but her attendants were desired to rouse her frequently during the night. On the following morning she complained of much pain and confusion in her head, with numbness of her extremities, and her bowels were obstinately costive. A large blister was applied between the shoulders, and strong doses of purgatives were given, but without producing any discharge from the bowels. In the evening I ordered the abdomen to be well rubbed with salt and to be fomented, which speedily produced a copious discharge of dark-colored and very offensive fæces. The numbness of the lower extremities, which indeed almost surmounted to paralysis, and an inability to contain her urine continuing, sinapisms were applied to her feet, which seemed to be of service. She remained for some days in a very lethargic state, with pain in the head, dimness of sight, and a constipated state of her bowels. These were relieved by the use of purgatives, and she is now perfectly well.

---

*Effects of Laudanum taken in large quantities.*—Case 1. W. W. æt. 70, took by mistake two tea-spoonfuls of tinctura opii (150 drops) on two successive nights. The first dose gave him a very good night, and on the following day he said his head felt as if he had taken laudanum, and that he had a frequent desire to make water without being able to empty the bladder. The mistake was not discovered, for it was supposed both by himself and friends that he he had taken two tea-spoonfuls of tinct. camphoræ comp. which had been recommended for a spasmodic affection of the organs of respiration, with which he was afflicted. I was called to him about six o'clock in the morning after the second dose. He was then much flurried, his pulse very quick, considerable thirst, and frequent micturition. He had then discovered his mistake. He was recommended to drink freely of lemonade, orange juice, coffee, &c. and some magnesia and rhubarb were given, which in a short time moved the bowels. He gradually recovered without much drowsiness, and the spasmodic affection of the organs of respiration was completely cured.

Case 2. J. B. æt. 68, drank, out of a bottle which had been accidentally left in his room, it is supposed, two ounces of vinum opii, at four o'clock in the morning. He soon after talked a great deal, and sung, after which, at six o'clock, he became sleepy and breathed laboriously. At nine o'clock what he had done was discovered: he was immediately roused, and compelled to swallow two scruples of white vitriol, and soon after six grains of tartarized antimony. Sickness followed, and he threw up more than a pint of fluid slightly tinged with laudanum. Vinegar and coffee were frequently administered, and he was kept awake the whole day. At seven in the evening he had a very strong full pulse, was less drowsy than he had been during the day, but appeared to suffer much from irritation in the bronchiæ; which was supposed to arise from some vinegar that might have passed into the larynx while the attendants were forcing him to swallow that remedy. A brown mucus was occasionally expelled from the trachea, a circumstance from which this suspicion derived confirmation. Shortly after he fell into a sleepy state, from which he could not by any means be roused, nor could he be made to swallow any thing. His breathing was rather apoplectic, and he perspired considerably. His pulse became more feeble, with irregularity and occasional intermissions. Slight subsultus was perceived, but no general convulsions. He remained in this state till the evening of the third day, when he died. When he could no longer swallow, blisters were applied to his chest and between the shoulders, and sinapisms to the feet, and an enema thrown up with vinum aloes. A stool followed a second injection, and some urine was passed. There was no appearance, either in the head or stomach, when these parts were examined two days after death, that could be attributed to the laudanum; the bladder was full of urine.—*Lond. Med. Rev.*

---

*Effects of Ol. Terebinthinæ rectificatum in Cases of Intestinal Worms.*—Case 1. A lady, the wife of a distinguished philosopher, formerly a physician, but who has long relinquished practice, had for many years been subject to complaints which were considered to arise from intestinal worms. Strong purgatives were had recourse to for their expulsion, and it was only from the use of these that she obtained any relief. Among the fæces were coils of cylindrical bodies of a dark olive color, of various lengths, and about the size of a crow's quill. The longest shewn to me was three inches.

When dried, they contracted so as to break transversely in several places, and were of a deep red color. Neither head nor tail could be accurately distinguished, but, as the extremities appeared truncated, perhaps none of them were entire. Notwithstanding all this, and the absence of motion, the husband of the lady was firmly persuaded that they were organized, and resolved, after perusing the communication from Dr. Fenwick, in the 2d vol. of the Transactions of the Medico-Chirurgical Society, to administer the ol. terebinthinæ. Two ounces were accordingly taken on the morning of Feb. 23d, after a light supper of gruel. Three stools followed within two hours, when another ounce was taken and thrown up again. It was however repeated, and remained upon the stomach. In the course of the day she had two more stools, and during the night was purged and vomited violently. Scybala, with mucus, and the bodies conceived to be worms, but in confused masses, were observed in the evacuations. She soon recovered from the violent operation of the medicine, and remained free from complaint until March 7th, when, after much uneasiness in the direction of the arch of the colon, she had a costive stool, and with it puckered shreds of coagulable lymph, about half an inch in breadth, and five or six inches in length, were discharged. The puckers were in the longitudinal direction of the shreds. None of the cylindrical bodies accompanied this evacuation. Castor oil was administered, which brought away more of the same kind of shreds. It has been repeated occasionally since, and she has at present no pain, nor can either the shreds of coagulable lymph or cylindrical bodies be now perceived in the fœces, neither has she any of the symptoms formerly attributable to worms.

Case 2. A lady, who had for several years been afflicted with ascarides, was advised to throw up two ounces of oil of turpentine with one ounce of warm milk, which she did several times, and remained free from the complaint ten months. About a month ago she threw up four ounces of the oil, with the same quantity of warm milk, and is now free from complaint. In the first proportion it occasioned much pain; and, having heard that it had been swallowed with safety in its pure form for other kinds of worms, she attempted to use it undiluted, but the pain occasioned by it was so great that she was compelled to desist.

Case 3. A little girl, six years of age, had for more than a year been troubled with ascarides. A table-spoonful of oil of turpentine with a small tea-cupful of warm milk, was thrown up, and repeated several times. This happened two years ago, and she has been free from the complaint ever since.

---

*Case of extensive Ossification of the semilunar Valves of the Aorta.*—Edward Giles, aged 60, had for some years complained of difficult respiration, of which the paroxysms were more severe during the winter season. He had a slight cough with occasional expectoration and constant pain at the scrobiculus cordis. The pulse was then very small, and sometimes irregular. In the winter of 1810, he was confined for many weeks: the dyspnœa was distressing tressing: the pain was constant, and he had violent palpitations of the heart. His bowels were irregular; his tongue covered with a white fur, and the upper part of his body generally bedewed with a cold perspiration. Some relief was obtained by large doses of opium and purgatives. In the beginning of July, 1811, he was attacked more severely. His pulse was so small as to be scarcely distinguishable, and could seldom be felt in his left arm. His respiration was convulsed, and he complained of great pain in the region of the heart. There was an evident pulsation in the epigastrium, synchronous with the pulse, and to this part he referred his pain. These symptoms increased till his pulse was imperceptible; the pulsation in the epigastrium had subsided, and his respiration was convulsed and at long intervals. He became anasarcous, particularly in the lower extremities, and suffered the most excruciating pain in the region of the heart. On the 28th of August he died. Dissection exhibited the following appearances: Both sides of the chest contained about a quart of bloody serum, and there was a large quantity in the abdomen. The cavity of the pericardium was very dry. The lungs were healthy. The heart appeared very large and distended with blood. An extensive ossification at the origin of the aorta could be distinctly felt externally. The semilunar valves were entirely converted into a bony substance, irregularly deposited and projecting into the cavity of the vessel so as to diminish its area to a remarkable extent. The opening which remained would not admit a very small quill, and was considerably less in diameter than the carotid artery of the same subject. The cavity of the left ventricle was considerably enlarged, and its parietes thickened. The right side of the heart and left auricle were very large and filled with blood. The surface of the heart was covered with white patches. The coronary arteries and ascending aorta were healthy, but the descending aorta was extensively ossified. The abdominal viscera were healthy.—*Lond. Med. Rev.*

---

*Case of Hernia Umbilicalis, operated on with Success during Utero-gestation. By John Taunton, esq. Surgeon to the City and Finsbury Dispensaries, and to the City Truss Society, Lecturer on Anatomy, Surgery, Physiology, &c.*—June 29, 1811. Mrs. E. P. aged 42, three months gone with child, has had an umbilical hernia for twenty years, as large as a full-sized teacup, but never found any inconvenience from it till yesterday, when, as she was walking in the street, a boy ran against her, and struck her upon the tumor, which occasioned much pain. A neighbouring surgeon and apothecary, who attended the family, was requested to see her. She had sickness, vomiting, hiccough, and constipation of the bowels; the pain, attended with a sensation of heat, was principally referred to the region of the stomach. Aperients, opiates, and enemas, were given; the former were rejected by the stomach, and the latter produced no effect on the bowels: fomentations were applied to the abdomen without relief. These means were pursued without benefit, and the symptoms increased till the morning of the 2d of July, when I was requested to see her.

 The

The operation was immediately recommended, and acceded to on her part. Indeed, I considered that too much delay had already taken place.

The integuments over the hernia were very thin, and in close contact with the peritoneal sac, which was also very thin, and every where in contact with, and adhering firmly to, the omentum. The omentum was partly torn through, and partly dissected from, the peritoneal sac, and a considerable portion of it was removed before the convolution of intestine was brought into view. The omentum was much inflamed, and in places approaching to a dark green color. The convolution of intestine was very dark, nearly in a state of gangrene. The stricture was very deep and firm; but when dilated there was not any impediment to the return of the bowel: nearly the whole of the omentum contained in the sac was removed. The extent of the inflammation on the omentum and intestine, the latter appearing almost in a state of gangrene, induced me to observe to the gentlemen who had been attending the case, that I was fearful that too much time had been lost, and that it was of the utmost importance to the safety of the patient, that the operation for hernia should be always performed early.

July 3d. She had a natural stool, was free from fever, and continued to recover without any unfavorable symptoms. The wound was completely healed in a month, and she was as well as usual, and continued so till her labor came on at the usual time. The hernia then protruded, and became strangulated; she had sickness, hiccough, vomiting, and constipation. She continued in this state for two days, when she was safely delivered. The hernia was still irreducible, and the vomiting and constipation continued till the next day, when the tumor lessened and the symptoms were relieved. At times she has had violent pains in the part, and the tumor has become tense and irreducible for a short period; but these symptoms have generally subsided without any surgical assistance. She could never be prevailed upon to wear a proper truss, though strongly recommended so to do.

April 20, 1812, four P.M. I was requested by her husband to visit her on account of her old complaint, of which she was now very ill. From his representation of the case I took my instruments, and Mr. Burn to assist at the operation should it be found necessary, in order that no time should be lost. We called upon the medical gentleman who had the care of the patient in this as well as in her former illness, to learn the history of the case now, and to have his assistance; but he was from home, having left a message that I was to do what I pleased with her. On his assistant referring to his day-book, it appeared that she was taken ill with symptoms of strangulation on the 16th, and visited by this gentleman on the 17th, when the symptoms were very urgent, and the hernia irreducible.

℞. Mag. sulphat. ℥ſs.

Mist amygdal. ℥vj. M. Fiat mist. Cochl. iij. 4tis horis.

On the 18th the symptoms had not been at all relieved, and she was worse in every respect. The following mixture, which she has been in the habit of taking subsequent to the former operation, was sent.

℞. Magn.

℞. Magn. sulphat. ℥j.

Mannæ ℥jſs.

Aq. menthæ ℥vij. M. Cochl. iij. 4tis horis.

A common glyster with one ounce of salts was now administered without any good effect.

On the 19th all the bad symptoms were aggravated: another glyster of the same kind was administered, and some castor oil which she had in the house was taken. She appeared at this time much worse; the castor oil was taken in coffee in the evening, and remained on the stomach during the night.

On this morning (four A.M. 20th) the skin on the lips, hands, and feet, and on the hips and loins, was discolored, rather purple: another glyster was administered, and three pills composed of jalapæ ℈j. Hydr. submuriat. gr. x. were taken, and immediately rejected by the stomach. Stercus-matter was now vomited, and all the symptoms indicated the greatest danger. Yet it does not appear that any means were taken to endeavor to obtain relief till four P.M. when I was first applied to, and visited her immediately.*

The pulse was low, weak, and irregular, at times the beat scarcely perceptible; cold clammy perspirations partially diffused over the body; the extremities cold, and of a very dark color: the skin on the face was nearly purple, and on the lips almost black; her strength was so much reduced, that she was not able to turn herself or to raise herself in bed without assistance.

The tumor was rather of a livid color, not very tense, but had that peculiar feel as if it was filled with fæces from a rupture of the intestine. There was but little pain in the hernial tumor, or on the lower part of the abdomen; but above, and rather inclined to the left side, there was a small part more tense and painful on pressure.

After stating the extreme danger in which she was, both to herself and to her husband, I named the operation as the only thing to be done, which could give any chance for her life, and left it for them to decide; which they did in the affirmative.

The incision was made in the direction of the linea alba, through the integuments, which were very thin, and adhered in every part to the peritoneal sac. There were about 3 oz. of a straw-colored fluid contained in the sac. The intestine was now exposed, being of a dark color, almost black. The peritoneal coat was abraded or ulcerated on one part to the extent of the size of a shilling: the stricture was between three and four inches deep, requiring the whole length of the bistoury: it was dilated a little both above and below, in the direction of the linea alba: there was no adhesion or impediment to the return of the intestine, nor was any force required. When the stricture was dilated, it appeared almost to withdraw itself into the abdomen.

The edges of the wound were laid together, and supported by strips of adhesive plaster and a compress of lint; the whole confined by a common roller.

* The above particulars were obtained from her husband, children, and herself, and also corroborated by the testimony of the assistant of the gentleman who had attended the case.

The

The following medicine was ordered, and two table spoonfuls directed to be taken every second hour.

℞. Conf. arom. ʒij.

Spt. lavd. comp. ℥jſs.

Tinct, opii ʒj:

Aq. menth. pip. ℥vjſs. M.

On visiting her at nine, P.M. four hours after the operation, the sickness and vomiting had entirely subsided. She had taken three cups of tea, which she seemed to like. She was still very cold, the clammy perspirations were not abated, and the skin was almost as much discolored as it was before the operation: but on the whole she felt herself relieved. The medicine was directed to be continued. She died at four o'clock in the morning, eleven hours after the operation.

It has often fallen to my lot to record similar cases; and it is with no small regret that I relate them, and that I see no prospect of their number being diminished, not even in London. Although here the physician can *without delay* have the advice of his colleagues, the surgeon the *immediate* assistance of his professional brethren, and the apothecary may have the advice and assistance of both physician and surgeon *almost instantly;* yet that wilful perverse desire of pursuing a routine of unsuccessful practice, of keeping the patient from proper assistance, for the purpose of making merchandise of his maladies, by the sale of a few medicines, still prevails, and is equally disgraceful to the profession, as it is almost certainly fatal to the patient. In the instance before us, a valuable parent was snatched from a *numerous offspring*, surely hurried to an untimely grave by a want of that assistance *which had been successful in a former case*, and which would in all human probability have been again completely successful, had it been applied in proper time.

The operation for hernia is simple, and, when performed with care, in proper time, is almost certainly successful. "Few, if any, would be the fatal cases of this operation, if it was performed sufficiently early."* The result of many years' practice, in which the number of operations on persons afflicted with this malady has been very considerable, enables me to speak with well grounded confidence on the success of this operation.

---

*Further particulars of the Case of an Affection of the Brain,*† *by Mr. Burne.*—A very short time after the first account of the case was published, the patient had a relapse. He had continued getting better until the 22d of February, when, without any apparent cause, he was taken so ill as to be obliged to retire to bed. Mr. Burne being sent for, found him remarkably weak: he spoke with difficulty, and articulated very badly; lay moaning in bed, and seemed to take little notice of any thing. The only symptom he complained of was a dull pain all over the head; the pupils of the eyes were dilated, but readily contracted on the application of a lighted

* Hey's Surgical Observations.
† Vide Medical and Physical Journal, vol. xxvii. p. 521.

candle.

candle. He ate little or nothing; could obtain no sleep; was very watchful; and would remain a whole day without asking for any thing or speaking to any one. His bowels were confined, and his stools black; the skin was hot and dry, and his tongue furred. The following powder was immediately ordered:

℞. Hydrargyri Submuriatis gr. vj.
Pulveris Antimonialis gr. ij. Misce.

Feb. 23.—The powder occasioned some dark-colored offensive evacuations. He still keeps moaning; is very thirsty, and his skin is hot and dry.

℞. Hydrargyri Submuriatis gr. j.
Pulveris Antimonialis gr. ij.
Nitratis Potassæ gr. x. Misce fiat pulvis quartis horis Sumendus ex melle.
His drink to be toast and water.

Feb. 24.—His bowels keep regularly open, but the fœces are black; his urine is small in quantity, high-colored, and, as he terms it, very hot; his pulse is regular, beats moderately firm, but by no means strong, or very full; his face is pale, and there is no appearance of congestion in the head. The symptoms are not in the least mitigated. The powders to be continued three times in the day.

Feb. 28.—His mouth is sore from the mercury; the saliva is considerably increased; he voids more urine; and the skin is moist, and bedewed with a gentle perspiration. His bowels are regular, and the evacuation not so black. His eyes are inflamed, and the light is painful to them. He takes a little gruel, but cannot sleep, though he is drowsy and anxious for sleep.

The powders to be discontinued, and the following mixture taken:

℞. Liquoris Ammoniæ Acetatis ℥ij.

Misturæ Camphoræ ℥vſs.

Syrup Croci ℥ſs. Misce. cochlearia duo ampla quartis vel sextis horis sumat.

March 3.—The inflammation of the eyes has quite subsided, and the light can be admitted without producing pain or inconvenience; he now begins to speak spontaneously; had several hours' sound sleep last night, and appears much refreshed. He is able to sit up in bed, and take broth, and small portions of the whiter kinds of meat, but is much reduced in bulk and strength. The bowels are regular, and the color of the fœces improves. Mixture to be continued. From this time to the 12th of March he continued rapidly recovering, and was then in all respects, except the articulation, quite well.

Mr. Burne conceives that the preceding symptoms were caused by pressure on some part of the brain. In the treatment he had three points in view: to regulate the evacuations of the alimentary canal, correct the morbid secretion of the bile, and keep up a gentle action or perspiration on the skin. The success of this treatment was evident; for, as soon as the skin became in a natural state, and the excretions of the bowels regular, and of a good color, so soon did the symptoms begin to be mitigated. This case then (as well as many others which might be adduced) proves the great importance and necessity of paying strict attention to the state of the skin, and of the alimentary canal, in *every disease*.

Mr. Chamberlaine having received very ample encouragement to proceed with his Tirocinium Medicum, on the duties of youth apprenticed to the medical profession; that work is now at press, and the printer gives hopes that it will be ready for delivery on the 1st of August. The price to subscribers to be five shillings, or at farthest not to exceed six shillings and sixpence. No money required to be paid until the delivery of the book. Subscribers names will be received by the author, at his house, No. 29, Aylesbury-street, Clerkenwell, and by all the medical booksellers.

